# Detecting cognitive motor dissociation by functional near-infrared spectroscopy

**DOI:** 10.3389/fneur.2025.1532804

**Published:** 2025-04-01

**Authors:** Yan Wang, Wentao Zeng, Leyao Zou, Qijun Wang, Bingkai Ren, Qi Xiong, Yang Bai, Zhen Feng

**Affiliations:** ^1^Affiliated Rehabilitation Hospital, Jiangxi Medical College, Nanchang University, Nanchang, Jiangxi, China; ^2^Rehabilitation Medicine Clinical Research Center of Jiangxi Province, Nanchang, Jiangxi, China; ^3^Key Laboratory of Jiangxi Provincial Health Commission for DOC Rehabilitation, Nanchang, Jiangxi, China; ^4^Center for Cognition and Brain Disorders, The Affiliated Hospital of Hangzhou Normal University, Hangzhou, Zhejiang, China; ^5^The First Affiliated Hospital, Jiangxi Medical College, Nanchang University, Nanchang, Jiangxi, China

**Keywords:** disorders of consciousness, cognitive motor dissociation, functional near-infrared spectroscopy, motor imagery, support vector machine

## Abstract

**Background:**

Behavioral assessment based on external manifestations of consciousness fails for patients with cognitive motor dissociation (CMD). Functional near-infrared spectroscopy (fNIRS) is an emerging neuroimaging technique that can detect internal brain functional activities. However, the extent to which fNIRS can help identify CMD patients among those with disorders of consciousness (DOC) remains unclear.

**Objective:**

To identify CMD patients among DOC patients using fNIRS with a command-driven hand-open-close motor imagery task.

**Methods:**

fNIRS was used to measure the hemodynamic responses of 70 prolonged DOC patients, including 30 with vegetative state/unresponsive wakefulness syndrome (VS/UWS), 20 with minimally conscious state minus (MCS–), and 20 with minimally conscious state plus (MCS+), during a command-driven hand-open-close motor imagery task. Seven features of hemodynamic responses were extracted during the task and the rest conditions. The support vector machine combined with genetic algorithm was employed to classify and predict the brain's response to spoken commands and to identify CMD patients among prolonged DOC individuals.

**Results:**

We identified seven CMD patients using fNIRS, of whom four were in VS/UWS and three were in MCS–. Six months after fNIRS examination, the seven identified CMD patients were more likely to have a favorable outcome (3/4 vs. 1/31, *P* = 0.014, Fisher's exact test) compared with non-CMD patients.

**Conclusions:**

CMD patients can be identified through fNIRS combined with a command-driven motor imagery task, which will aid in the accurate diagnosis of DOC patients.

## Introduction

Disorders of consciousness (DOC) are conditions characterized by changes in arousal and/or awareness resulting from severe brain injury or disease (1), including coma, vegetative state/unresponsive wakefulness syndrome (VS/UWS), and minimally conscious state (MCS) (1, 2). Coma is defined as a state with a complete lack of arousal (eyes closed) and awareness (3). VS/UWS is defined as a state of preserved arousal (eyes open) but without awareness (4). While MCS is defined as the minimal, reproducible, but inconsistent state of awareness (5), it can occur without (MCS–) or with (MCS+) evidence of language function (6). The DOC lasting more than 28 days is called prolonged DOC (7). Accurate assessment of the level of consciousness in DOC patients is crucial for treatment planning, rehabilitation programs, and prognosis prediction. Currently, subjective behavioral assessments (Coma Recovery Scale-Revised, CRS-R; Full Outline of Unresponsiveness, FOUR, etc.) are commonly regarded as clinical reference standard for assessing the level of consciousness in DOC patients (8). However, these behavioral assessments rely on external behavioral manifestations of consciousness, and may fail when consciousness cannot be expressed through external behaviors. Accurate assessment of the conscious state in DOC patients is complex and challenging.

Fortunately, brain activities are accompanied by changes in bioelectrical activities, cerebral blood oxygen levels, and brain metabolism. Neuroimaging and electrophysiology techniques used to detect these representations provide us an access to objectively detect brain activities and subsequently assess the consciousness level in DOC patients. In 2006, researchers verbally instructed a patient who met the behavioral criteria of VS/UWS to perform two mental imagery tasks (imagining playing tennis and imagining visiting her own house) during the functional magnetic resonance imaging (fMRI) scan (9). The patient exhibited neural responses similar to those of healthy volunteers when performing the same imagination tasks, indicating the patient had residual cognition. Subsequently, these researchers conducted fMRI scans of mental imagery tasks on 54 patients, where five cases of behaviorally unresponsive patients with VS/UWS and MCS showed meaningful brain activation (10). Similarly, in another study, electroencephalogram (EEG) recordings revealed 27 cases of brain activation in response to spoken commands among 193 DOC patients with acute brain injury who were unable to follow spoken commands (11). This led to the proposal of the concept of cognitive motor dissociation (CMD) (12), specifically referring to the patients who are behaviorally unresponsive (coma, VS/UWS, and MCS–) but have neuroimaging and electrophysiology evidence of command following. Conversely, the other behaviorally unresponsive patients without neuroimaging and electrophysiological evidence of command following are called non-CMD patients (13, 14), or true DOC patients, as defined by the Motor Behavior Tool (15). Recently, 2020 European Academy of Neurology guidelines suggested that task-based neuroimaging and electrophysiology should be used where feasible to accurately detect consciousness in DOC patients (16).

Functional near-infrared spectroscopy (fNIRS) is a recently emerged neuroimaging technique based on optical principles (17). By measuring differential absorption of near-infrared light with a wavelength of 600–900 nm by oxyhemoglobin (HbO) and deoxyhemoglobin (HbR) in brain tissue, fNIRS can detect hemodynamic changes in the cerebral cortex, and indirectly detect functional activities based on the law of neurovascular coupling (17, 18). In comparison to fMRI, fNIRS is less susceptible to motion artifacts and metal implants and offers greater temporal resolution (8, 17, 18). It can simultaneously measure changes in the concentrations of HbO, HbR, and total hemoglobin (HbT), providing more comprehensive information to better characterize hemodynamic responses (8). Furthermore, fNIRS is cost-effective, portable, convenient, and can be used for repeated bedside monitoring of the brain function of DOC patients (8, 18). Compared to EEG, fNIRS has superior electromagnetic compatibility and spatial resolution (8, 18).

As an emerging brain functional imaging technique, researchers first proved the feasibility of using fNIRS combined with an imagined squeezing a ball task to assess brain function in DOC patients in 2016 (19). Subsequently, researchers successfully communicated with a functionally locked-in patient using fNIRS combined with an imagined playing tennis task (20). A study further revealed that three out of five MCS (2MCS+ and 1MCS–) patients exhibited hemodynamic responses similar to those of healthy controls (HC) during an imagined playing tennis task (7). However, studies on brain functional activities of DOC patients and assessment of consciousness state using fNIRS are still scattered and limited. Currently, there are no reports on fNIRS specifically targeting CMD patients from DOC patients without command-following abilities (coma, VS/UWS, MCS–). Identifying CMD can capture the internal cognition of these patients, improve care, and guide treatment and prognosis (11). In this study, we used fNIRS to identify CMD patients from DOC patients. Additionally, we explored the utility of fNIRS in the diagnosis of DOC.

## Materials and methods

### Participants

In this study, 70 DOC patients were recruited from the Department of Rehabilitation Medicine at the First Affiliated Hospital of Nanchang University. The inclusion criteria were: (i) patients aged 16 to 80 years; (ii) the duration of DOC lasting more than 28 days; (iii) right-handedness; (iv) complete auditory brainstem evoked potentials confirmed by electrophysiological examination; and (v) providing informed consent. The exclusion criteria were: (i) patients with unstable vital signs; (ii) patients with cranial defects who underwent debridement flap decompression; (iii) patients with alcohol or substance abuse or a previous history of neurological or psychiatric disorders; (iv) patients with a large amount of intracranial hematomas impacting data quality; (v) patients with scalp injuries preventing skullcap wear; and (vi) patients who had received sedative medication within the past 24 h. In addition, 70 healthy right-handed individuals were recruited as controls. An informed consent form was signed before the start of the study, and the purpose, risks, procedures, and significance of the experiment were explained in detail. Patients' legal guardians provided written informed consent, and HC provided consent for themselves. The study adhered to the Declaration of Helsinki and obtained approval from the Ethics Committee of the First Affiliated Hospital of Nanchang University (approval number IIT2023-222).

### Experimental paradigm

**Figure 1:**
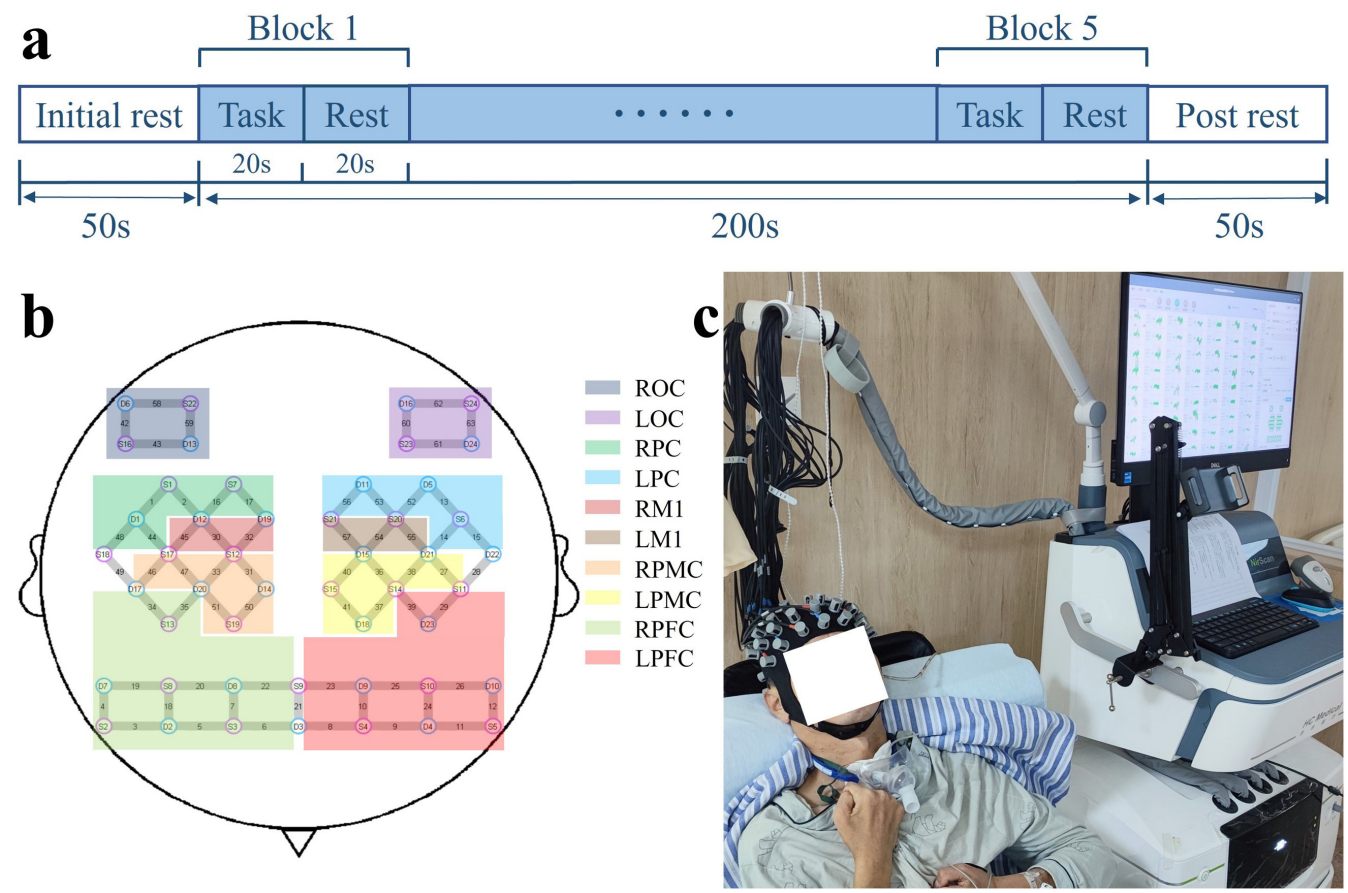
**(a)** Experimental paradigm of the motor imagery task. **(b)** Location of probes and channels and delineation of regions of interest. **(c)** The patient is undergoing an fNIRS examination. ROC, right occipital cortex; LOC, left occipital cortex; RPC, right parietal cortex; LPC, left parietal cortex; RM1, right primary motor cortex; LM1, left primary motor cortex; RPMC, right premotor cortex; LPMC, left premotor cortex; RPFC, right prefrontal cortex; LPFC, left prefrontal cortex.

Both DOC patients and HC were required to perform a hand-open-close motor imagery (MI) task (21). Throughout experiment, the hemodynamic responses of cerebral cortex of all participants were detected by the fNIRS system. During the task, participants were asked to imagine repeatedly opening and closing their both hands as quickly and naturally as possible without distinguishing between the left and right. This relatively simple task paradigm reduces the cognitive processing demands (22). We believe that MI of both hands is more conducive for the performance of DOC patients. The MI task was designed in an experimental block paradigm, consisting of a 20-s imagery task followed by a 20-s rest. The block paradigm condition was repeated five times. Because auditory pathway is a relatively well-preserved information input channel in DOC patients (23), each condition command (“imagery” and “rest”) was presented verbally. A scheduled procedure was triggered to alert participants when the task began or when the rest period started. A 50-s pre-baseline and post-baseline period preceded and followed the repetition of the block paradigm, allowing participants to remain at rest so that cortical hemodynamics could stabilize at baseline levels. The total duration of the experimental paradigm was 300 s (see Figure 1a). The Glasgow Outcome Scale-Extended (GOSE) scores of the patients were followed up 6 months after fNIRS examination.

### fNIRS data acquisition

Cortical hemodynamic activity of each participant was recorded using a continuous-wave fNIRS system (NirScan-6000A, Danyang Huichuang Medical Equipment Co., Ltd., Jiangsu, China). The fNIRS system utilizes wavelengths of 703 nm, 808 nm, and 850 nm, with a sampling frequency of 11 Hz. Referring to the international 10–20 EEG electrode placement system, 24 source optodes and 24 detector optodes were symmetrically placed in the frontal, parietal, temporal, and occipital lobe areas via a flexible headgear, with a spacing of 3 cm, forming 63 measurement channels (see Figure 1b). Space registration was based on a standard head model. Specifically, spatial positions of the source optodes, detector optodes, and anchor points (located at Nz, Cz, Al, Ar, and Iz, based on the international 10–20 EEG electrode placement system) were measured using a 3D electromagnetic digitizer (Patriot, Polhemus, USA). The acquired coordinates were converted to Montreal Neurological Institute (MNI) coordinates in NirSpace (Danyang Huichuang Medical Equipment Co., Ltd., Jiangsu, China), and subsequently projected onto the MNI standard brain template. Brodmann areas were then determined using a space registration method (24). Supplementary material 1 shows the MNI coordinates for each channel and optode. Supplementary material 2 shows the Brodmann areas for each channel and optode. Based on the above information, 10 regions of interest (ROI) were delineated: left prefrontal cortex (LPFC) (channels #8–12, 23–26, 29, 39); right prefrontal cortex (RPFC) (channels #3–7, 18–20, 22, 34, 35); left premotor cortex (LPMC) (channels #27, 36–38, 40, 41); right premotor cortex (RPMC) (channels #31, 33, 46, 47, 50, 51); left primary motor cortex (LM1) (channels #54, 55, 57); right primary motor cortex (RM1) (channels #30, 32, 45); the left parietal cortex (LPC) (channels #13–15, 52, 53, 56); right parietal cortex (RPC) (channels #1, 2, 16, 17, 44, 48); left occipital cortex (LOC) (channels #60–63); right occipital cortex (ROC) (channels #42, 43, 58, 59). Figure 1b shows the delineation of the regions of interest.

Data acquisition was performed in a quiet room with soft lighting. Patients were positioned comfortably in a wheelchair, and data were collected while they were awake. HC were asked to maintain a good mental state, refrain from smoking, drinking alcohol or coffee, taking psychotropic medication, or engaging in strenuous exercise prior to data collection. They were tested in a comfortable sitting position. Following the wearing instructions, both patients and HC were fitted with a headgear containing optodes (see Figure 1c). It was ensured that the optodes were securely attached to the scalp. Where necessary, hair was swept away or cut to maximize the efficiency of light coupling to the tissue. Prior to data collection, both patients and HC were informed in detail about the experimental paradigm and were asked to minimize head movements. They also underwent simulation training to enhance task performance. When all preparations were completed, the fNIRS system was used to gather brain functional data during the MI task from both patients and HC.

### fNIRS data processing and feature extraction

NirSpark software (Danyang Huichuang Medical Equipment Co., Ltd., Jiangsu, China) was used to preprocess fNIRS signals (17, 25, 26). Firstly, an experienced data analyst performed an initial data check and excluded any poor-quality data. The signal-to-noise ratio for each channel was then calculated using the coefficient of variation (CV = σ/μ × 100%, where σ represents the standard deviation and μ represents the mean value of the signal), discarding channels with CV >15% (27). The remaining data underwent the following processing steps: (i) Converting raw light intensity to optical density. (ii) Utilizing a sliding window [with a threshold standard deviation of six and a threshold amplitude of 0.5 (25)] combined with cubic spline interpolation to identify and correct motion artifacts caused by head movements, etc. (25, 26, 28). (iii) Removing interference signals caused by heart rate, breathing rate, Mayer waves, and low-frequency signal drift using 0.01 to 0.1 Hz band-pass filtering (8, 25). (iv) All differential path-length factors were set to 6.0 and the relative concentration changes in HbO and HbR of each channel were calculated according to the modified Beer-Lambert law (25).

After preprocessing, average hemodynamic responses within the 10 ROIs were calculated. Data was then segmented into epochs from 5 s before each block to the end of each block (−5 s−40 s). After baseline correction (the data of 5 s before each block [−5s−0s] was used as baseline), block-averaged hemodynamic responses were calculated. Finally, seven commonly used fNIRS features—mean, peak, variance, median, peak-to-peak, skewness, and kurtosis (29–32)—were extracted from HbO and HbR responses during MI and rest periods. Features were specifically extracted 5 to 20 s following the onset of task and rest commands, accounting for the lag effect of hemodynamic responses (8, 33, 34).

### fNIRS data classification

**Figure 2:**
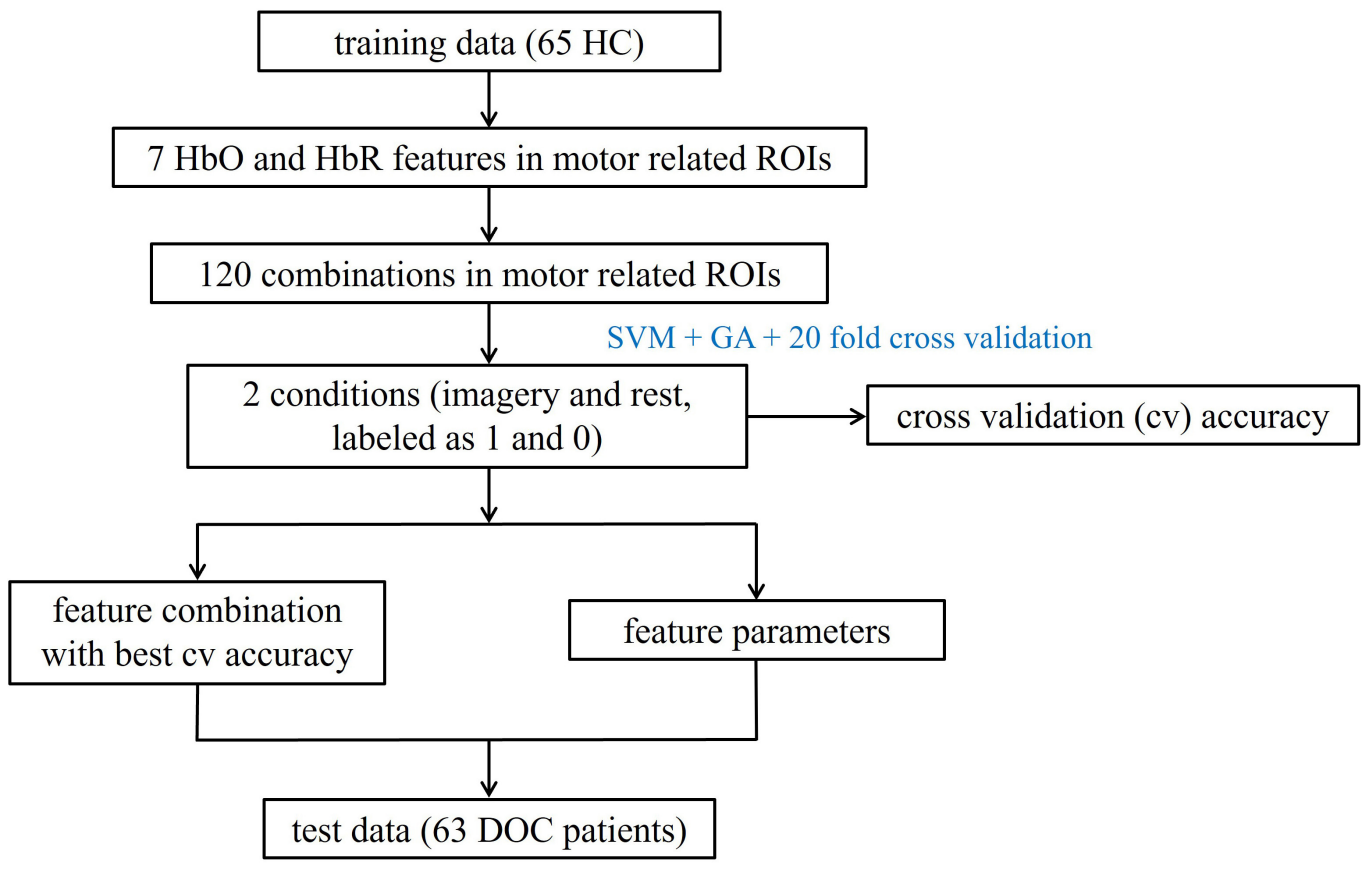
Workflow for fNIRS data classification. HC, healthy controls; HbO, oxyhemoglobin; HbR, deoxyhemoglobin; ROI, regions of interest; DOC, disorders of consciousness.

For each ROI, we combined Support Vector Machines (SVM) with Genetic Algorithms (GA) to identify the optimal parameters C and γ. Specifically, HC were used for model training, as they can reliably follow commands and provide clear task response labels (imagery vs. rest), which helps establish a well-defined reference pattern for the MI task. We selected seven HbO and HbR features and explored the performance of 120 feature combinations (excluding one-dimensional features and forming combinations of 2 to 7 features out of the seven available) to classify the two conditions (35), labeled as 1 for imagery and 0 for rest. Optimal parameters for each combination were identified by GA based on the best 20-fold cross-validation accuracy. The combination of mean, variance, peak-to-peak, and skewness within motor function-related ROIs (LPMC, RPMC, RM1, LM1) showed the highest accuracy with their optimal parameters. Additionally, in healthy individuals, the general linear model confirmed the activation of motor-related brain regions during the MI process, as detailed in Supplementary Figure 1. In summary, classification models for these ROIs were built on a training set of 65 HC using optimal parameters and feature combinations, with 20-fold cross-validation assessing model robustness. The model was then applied to predict a test set of 63 DOC patients, allowing identification of fNIRS responses to MI tasks based on predicted labels (1 for imagery, 0 for rest). Figure 2 illustrates the workflow for fNIRS data classification. Data analysis was completed on the MATLAB (R2022a, The MathWorks, Inc., Natick, Massachusetts, United States) and the Libsvm-Faruto toolbox (Libsvm-Faruto Ultimate V3.1, https://github.com/faruto/Libsvm-FarutoUltimate-Version).

### Statistical analysis

Demographic differences between the training and testing sets were assessed using the independent samples *t*-test or Mann-Whitney U test for continuous variables, and the chi-square test or Fisher's exact test for categorical variables. To evaluate the reliability of fNIRS detection of command-following evidence in the MI task, we calculated the true positive rate (TPR; i.e., sensitivity), true negative rate (TNR; i.e., specificity), false positive rate (FPR), and false negative rate (FNR) within the patient cohort (36, 37). Behavioral diagnosis based on CRS-R served as the reference standard for command-following, while fNIRS response to MI task was utilized as the test standard (36). Then, a confusion matrix was constructed. The false positive patients (those without behavioral command following but exhibiting fNIRS response to the MI task) were the CMD patients we were searching for. Patient prognosis was dichotomized as favorable outcome [upper severe disability or better (GOS-E score of 4–8)] or unfavorable outcome [lower severe disability or worse (GOS-E score of ≤ 3)] (11, 38, 39). The prognosis of CMD patients and non-CMD patients was compared using the Fisher's exact test. The statistical analysis was completed with SPSS 26 software (SPSS Inc., Chicago, IL, United States).

## Results

### Demographics and clinical characteristics

FNIRS data from 70 prolonged DOC patients (30 VS/UWS, 20 MCS–, 20 MCS+) during MI tasks were successfully collected. Among them, data from seven patients (2 VS/UWS, 4 MCS–, 1 MCS+) were excluded from further analysis due to significant motion artifacts or a large coefficient of variation. Meanwhile, we collected fNIRS data from 70 HC, with five individuals excluded due to significant motion artifacts or large coefficient of variation. Ultimately, the training set included 65 HC, while the testing set comprised 63 DOC patients. Detailed demographic and clinical characteristics of the training and testing sets are presented in Table 1. There was no significant statistical difference in gender between the two datasets (χ^2^ = 1.596, *p* = 0.206); however, there was a significant statistical difference in age (U = 730.5, *p* < 0.001).

**Table 1:** Demographic and clinical characteristics of the training set and the testing set.

**Items**	**Testing set *n* = 63**	**Training set *n* = 65**	**Statistics**	***p*-value**
Gender			χ^2^ = 1.596	0.206^a^
male	40	48		
female	23	17		
Age (years)	60 (16)	44 (26)	U = 730.5	< 0.001^b^
Etiology				
traumatic brain injury	16	-	-	-
hemorrhagic stroke	36	-		
ischemic stroke	7	-		
anoxia	4	-		
Time from DOC onset to fNIRS (days)	55 (44)	-	-	-

Gender and etiology are presented as frequencies. Age and time from DOC onset to fNIRS are presented as medians (interquartile range). ^a^p-value was obtained using Chi-square test. ^b^p-value was obtained using Mann-Whitney U test.

### Finding CMD patients with MI tasks

**Figure 3:**
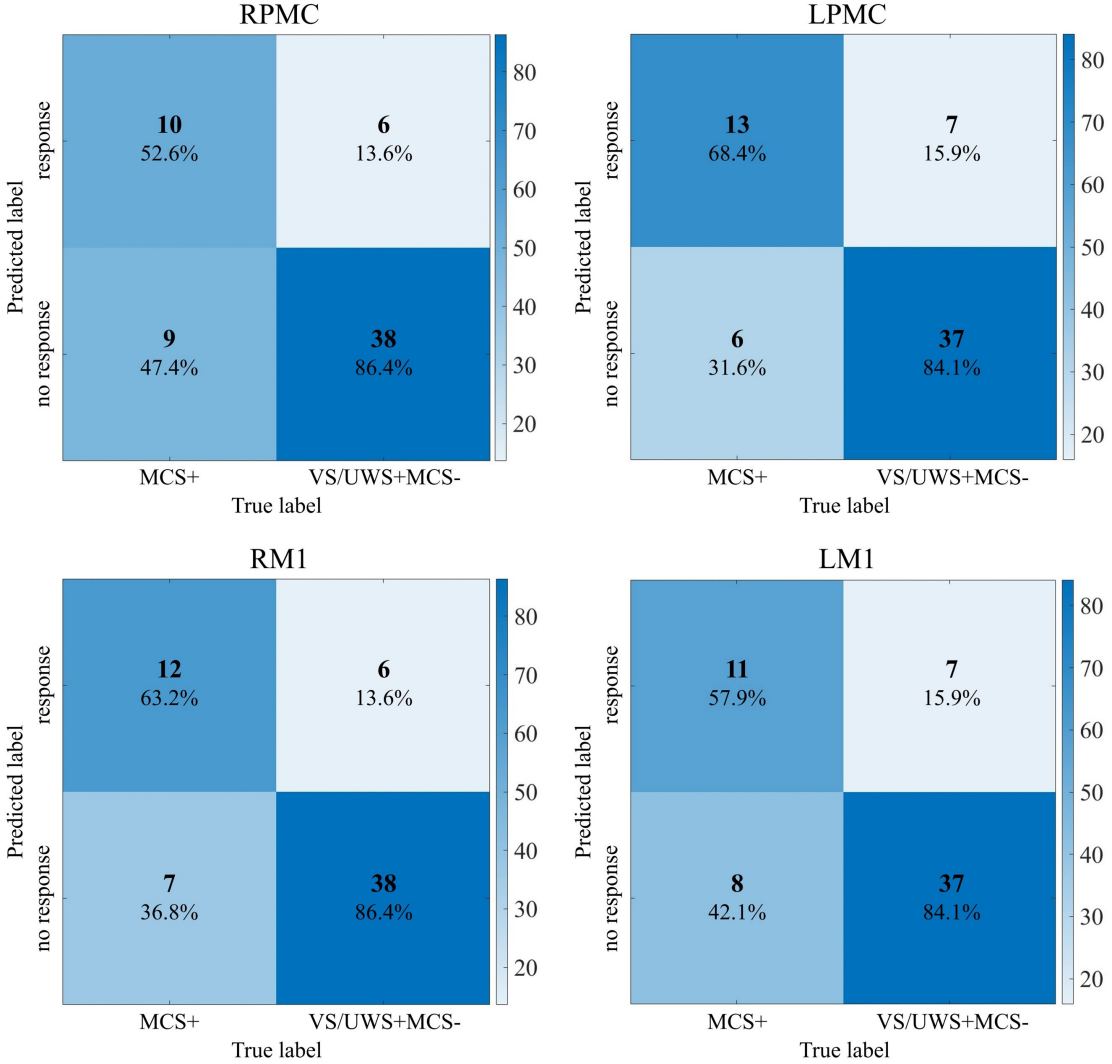
Confusion matrix of fNIRS response to MI task on 4 ROIs. RPMC, right premotor cortex; LPMC, left premotor cortex; RM1, right primary motor cortex; LM1, left primary motor cortex; VS/UWS, vegetative state/unresponsive wakefulness syndrome; MCS, minimal conscious state.

**Figure 4:**
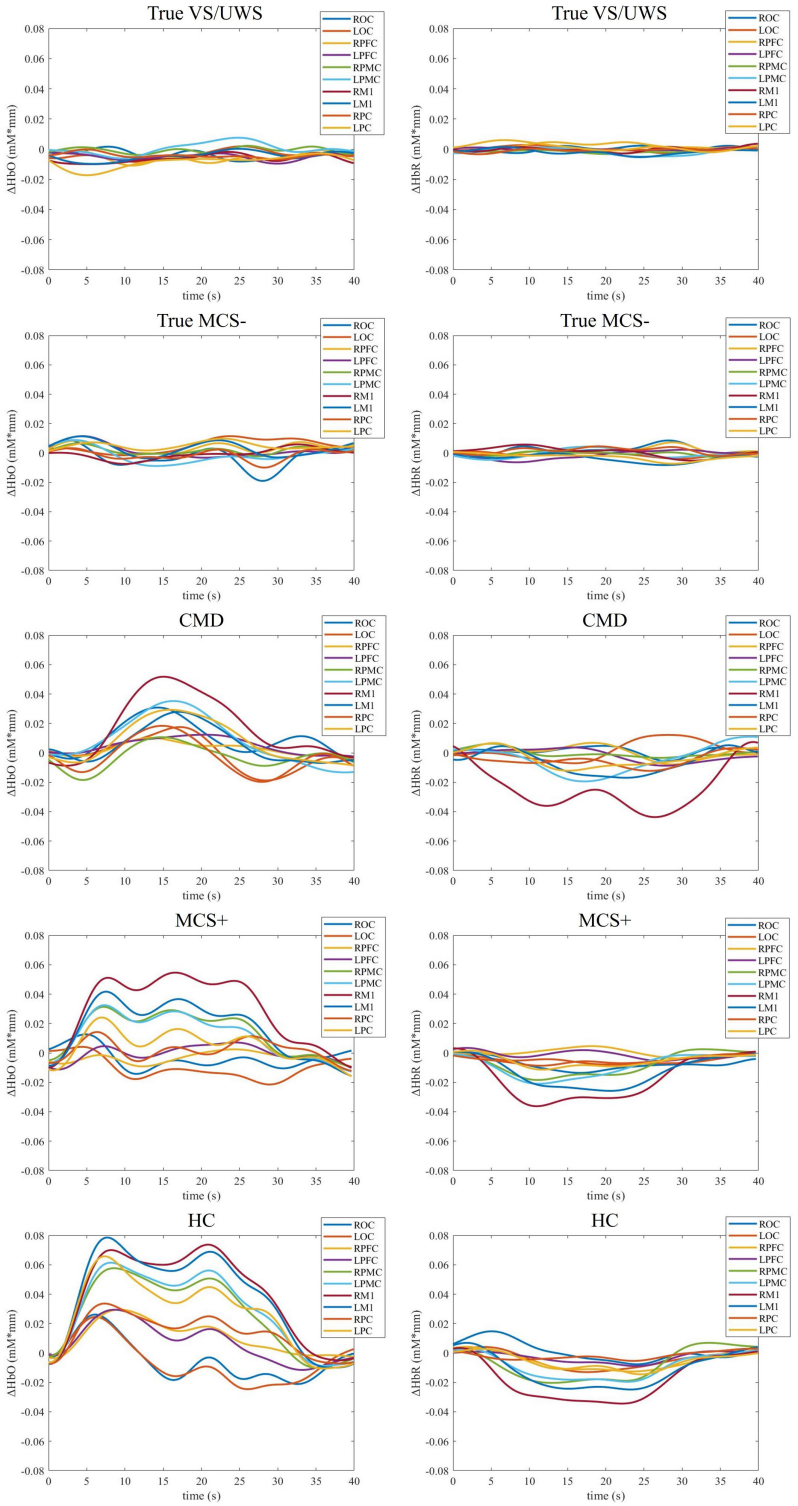
Task-evoked block-averaged hemodynamic response curves of one representative true VS/UWS patient, one true MCS– patient, one CMD patient, one MCS+ patient, and one HC. VS/UWS, vegetative state/unresponsive wakefulness syndrome; MCS, minimal conscious state; CMD, cognitive motor dissociation; HC, healthy control. ROC, right occipital cortex; LOC, left occipital cortex; RPC, right parietal cortex; LPC, left parietal cortex; RM1, right primary motor cortex; LM1, left primary motor cortex; RPMC, right premotor cortex; LPMC, left premotor cortex; RPFC, right prefrontal cortex; LPFC, left prefrontal cortex.

The combination of GA and SVM was used to construct a classification model and identify the response of patients with prolonged DOC to MI commands. In the training set, the model achieved a cross-validation accuracy of 95.4% (with parameters C = 6.69 and γ = 6.08) in the RPMC. In the LPMC, the accuracy was 91.5% (with C = 3.64 and γ = 7.66). In the RM1, the accuracy was 90.0% (with C = 0.21 and γ = 1.63). In the LM1, the accuracy was 93.1% (with C = 9.02 and γ = 9.80). The confusion matrices for identifying the response of patients with prolonged DOC to MI commands across the four ROIs (RPMC, LPMC, RM1, LM1) are shown in Figure 3. The highest sensitivity (68.4%) was observed in LPMC. Hence, the results from LPMC were considered as the final identification outcomes. Among the 19 MCS+ patients with command-following abilities, 13 showed fNIRS responses to the MI task [TPR = 68.4% (13/19), FNR = 31.6% (6/19)]. Among the 44 VS/UWS and MCS– patients without command-following abilities, 37 showed no fNIRS responses to the MI task [TNR = 84.1% (37/44), FPR = 15.9% (7/44)]. The sensitivity of fNIRS response to MI tasks for detecting patients' command-following abilities was 68.4%, and the specificity was 84.1%. In this context, seven CMD patients were identified, four from VS/UWS patients and three from MCS– patients. Therefore, among the VS/UWS and MCS– patients, the ratio of CMD patients was 15.9% (7/44). These CMD patients were missed by CRS-R evaluation, while the remaining 37 patients were true DOC patients. Figure 4 shows the block-averaged hemodynamic response curves of HbO and HbR for a true VS/UWS patient, a true MCS– patient, a CMD patient, an MCS+ patient, and a HC during the MI task. The typical hemodynamic response is characterized by a significant increase in HbO concentration along with a slight decrease in HbR concentration. The trends of hemodynamic response during the MI task in the CMD patient, the MCS+ patient, and the HC were found to be consistent.

### Prognosis of CMD patients

**Figure 5:**
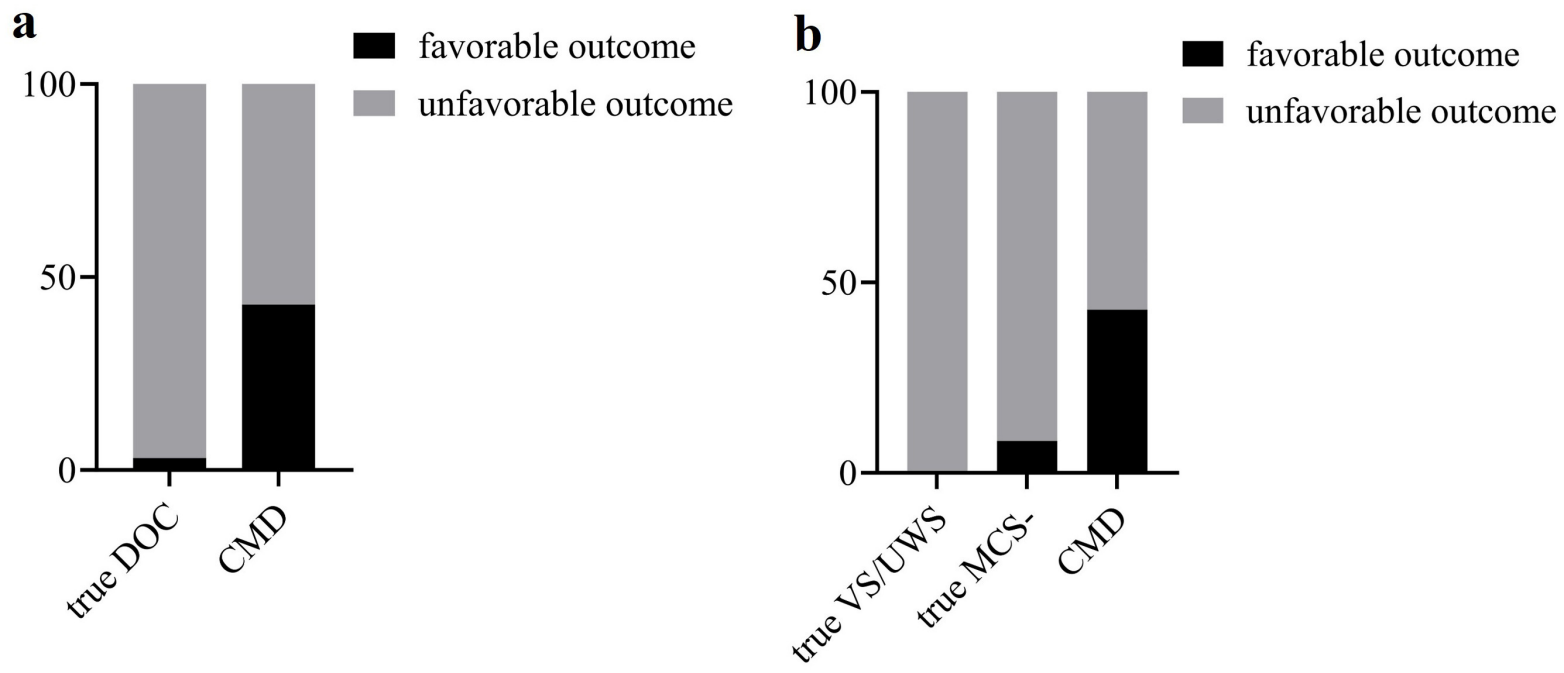
**(a)** Comparison of prognosis between true DOC patients and CMD patients. **(b)** Comparison of prognosis between true VS/UWS patients, true MCS– patients and CMD patients. DOC, disorders of consciousness; CMD, cognitive motor dissociation; VS/UWS, vegetative state/unresponsive wakefulness syndrome; MCS, minimal conscious state.

Six months after fNIRS examination, seven CMD patients were all followed up, while five were lost in 37 true DOC patients. CMD patients were more likely to have a favorable outcome (GOS-E score of 4–8) compared with true DOC patients (3/4 vs. 1/31, *P* = 0.014, Fisher's exact test, Figure 5a). However, among the seven CMD patients, there was still one with a GOS-E score of three and three with a GOS-E score of 2. Furthermore, intergroup differences in prognosis among CMD patients, true VS/UWS patients, and true MCS– patients were analyzed (Figure 5b). There was a significant statistical difference between true VS/UWS patients and CMD patients (0/20 vs. 3/4, *P*=0.012, Fisher's exact test). However, there was no significant statistical difference between true VS/UWS patients and true MCS– patients (0/20 vs. 1/11, *P*=0.375, Fisher's exact test), nor between true MCS– patients and CMD patients (1/11 vs. 3/4, *P*=0.117, Fisher's exact test). Multiple comparisons between groups were corrected using Bonferroni adjustment, with *P* < 0.0167 considered statistically significant.

## Discussion

Consciousness is a multifaceted concept. Medically, it signifies the state of patients' awareness of self and environment, as well as their responses to external stimuli and internal needs (40). The behavioral assessment, based on the external manifestations of consciousness, is the simplest and most intuitive approach, often regarded as the gold standard for assessing the level of consciousness (7). CRS-R has undergone continuous improvement and has become the most classic, detailed, and widely used behavioral assessment tool (41–43). It classifies DOC into coma, VS/UWS, MCS–, and MCS+ based on rigorous scoring criteria across six aspects. However, consciousness can only be captured and identified by behavioral assessment when it is expressed through external behavioral manifestations. This is the greatest drawback of behavioral assessment. In abnormal circumstances where consciousness cannot be externalized through observable manifestations, behavioral assessment becomes ineffective, leading to a significant underestimation of the actual state of consciousness in these patients. For such patients, it is terrible that their inner world (feelings, thoughts, desires) will be in the dark and cannot be known by doctors and family members. On the other hand, due to the subtlety, inconsistency, and volatility of consciousness in DOC patients, as well as the subjectivity of assessors (44), misdiagnosis of the level of consciousness in DOC patients is not uncommon, with misdiagnosis rates reaching up to 40% (8, 44).

Neuroimaging and electrophysiology techniques compensate for the shortcomings of behavioral assessments to some extent. They do not rely on external manifestations of consciousness or subjective judgments of assessors. They play an important role in objectively identifying patients' conscious states and exploring their inner world from the perspective of brain functional activities related to consciousness. In this study, we used fNIRS, an emerging neuroimaging technique, combined with the MI task to discern indications of consciousness in prolonged DOC patients. More specifically and importantly, we focused on finding evidence of consciousness activities in unresponsive DOC patients (coma, VS/UWS, MCS–). These patients are referred to as CMD patients (11, 12).

In this study, we adopted the hand-open-close MI task, requiring participants to perform the task with both hands without discriminating between left and right. We thought that intricate and advanced MI tasks, such as imagining playing tennis (7, 20, 45) and imagining playing badminton (8), might pose challenges for DOC patients to execute. More importantly, if participants lacked prior experience in playing badminton or tennis, it would impede the successful performance of the MI task. Therefore, we opted for a hand-open-close MI task. Performing with both hands without distinguishing between left and right further reduces the difficulty of the task and avoids the problem that unilateral structural brain injury may affect brain functional activities. We have made the greatest effort to detect brain functional activities. However, the MI task is an internal cognitive process, and the quality of the task is difficult to monitor, which may subsequently affect the data and results. For HC, incorporating actual execution of the motor tasks (rather than just imagining them) may help to compare and validate the quality of the MI tasks and assist in excluding some “poor imagers” from the dataset. This could be a viable option for consideration in the future.

Previous research has confirmed the applicability of SVM in classifying hemodynamic responses under different conditions (7). When using the mean and peak of HbO in the M1 and S1 areas as features, SVM achieved an 80% classification accuracy in healthy individuals. When the mean and peak of HbR were added as features, the classification accuracy was improved to 90% (7). This indicated that adding classification features can enhance classification accuracy, and HbR can also provide additional useful information for classification (7). In our study, we used seven features of HbO and HbR within 10 ROIs and combined SVM with GA. The global search ability of GA was utilized to find the optimal parameter combination and optimal feature subset of SVM. This approach was instrumental in enhancing the classification accuracy and robustness of the algorithm, while mitigating the risk of overfitting (46, 47). As expected, the classification accuracy of the model we constructed on the four ROIs (RPMC, LPMC, RM1, LM1) of HC was 95.4%, 91.5%, 90.0%, and 93.1%, respectively. Using this model, we successfully classified the MI and rest conditions of 13 out of 19 MCS+ patients, and detected their conscious activities in response to MI commands. Some researchers also used MI tasks and neuroimaging and electrophysiology techniques to detect consciousness (36). In MRI evidence, using traditional methods to quantify the percentage of activated voxels within the ROI and setting statistical thresholds, researchers discovered brain responses in 11 out of 16 healthy individuals (sensitivity of 68.8%) and in three out of seven patients with command-following (sensitivity of 42.9%). Their EEG findings revealed brain responses in 12 out of 16 healthy individuals (sensitivity of 75%) and in three out of nine patients with command-following (sensitivity of 33.3%) using SVM (36). We used healthy individuals to train the classification model, which was then applied to test DOC patients. By combining SVM with GA, we were able to increase the sensitivity of identifying the brain responses of MCS+ patients to 68.4%.

We found that LPMC exhibited the highest sensitivity. This finding may be attributed to the role of the premotor cortex in motor planning and preparation (48–50), along with the dominance of the left hemisphere. We believe that premotor cortex plays an important role in the hand-open-close MI task. Based on the identification results obtained from the LPMC, we identified seven CMD patients, and we found CMD patients were more likely to have a favorable outcome (GOSE≥4) than true DOC patients 6 months after fNIRS examination. More specifically, CMD patients had a better prognosis than true VS/UWS patients. This finding was consistent with previous reports (11, 14, 37), which further confirmed the reliability of machine learning recognition results. However, there was still one patient with a GOSE score of three and three patients with a GOSE of 2. Moving forward, we intend to continue monitoring the prognosis of these four CMD patients.

Using fNIRS, we explored the consciousness activities of prolonged DOC patients and successfully discerned the consciousness traces of seven unresponsive patients. This finding provides more evidence for the diagnosis of these patients beyond behavioral assessment. The diagnosis of CMD serves not only as a conclusion but also as a guiding factor for patient treatment and prognosis, while also providing valuable insights for the allocation of medical resources and ethical considerations (51–53). In a study involving 193 acute DOC patients lacking command following, Egbebike et al. (11) identified 27 CMD patients through EEG. In our study, we identified seven CMD patients among 44 prolonged DOC (VS/UWS and MCS–) patients using fNIRS. The prevalence of CMD among patients with acute (14.0%) or prolonged (15.9%) DOC was found to be comparable.

This study further proves the value of fNIRS in assessing the conscious state of DOC patients. However, there are some limitations. Firstly, we identified only seven CMD patients. The small sample size poses greater challenges in ensuring the robustness of our results and constrains the generalizability of our findings. It also affects the statistical power of patient prognosis. In the future, we aim to increase the sample size to enhance the generalizability of our findings and improve the statistical power. Secondly, similar to other neuroimaging and electrophysiology techniques such as fMRI and EEG (36), fNIRS also has technical defects in sensitivity. In our study, LPMC exhibited the highest sensitivity (68.4%), while the sensitivity of the other three ROIs was lower. That is, failure to detect brain functional activities following task commands does not mean that there is no consciousness. Sensitivity is a common challenge across all neuroimaging and electrophysiology techniques. The combination of multimodal neuroimaging and electrophysiological techniques (fNIRS, fMRI, EEG, etc. whether detected simultaneously or separately) may help improve detection sensitivity. Thirdly, the ages of training set and testing set do not match, which may have a potential impact on the dataset and the results. Finally, steep learning curve associated with fNIRS data analysis poses a barrier to widespread clinical application and adoption of this technique. Moving forward, additional research is needed to enhance the sensitivity of neuroimaging and electrophysiology techniques (by designing more sensitive paradigms or combining different neuroimaging and electrophysiology techniques). Software or programs should be developed to explore automated data preprocessing and analysis to assist clinicians in quickly obtaining results. We believe that repetitive bedside behavioral assessments combined with portable neuroimaging and electrophysiology techniques will facilitate a better assessment of the conscious state of DOC patients in the future.

In summary, fNIRS can objectively detect the consciousness of DOC patients from the perspective of brain functional activities. We found seven CMD patients with fNIRS, who were more likely to have a favorable outcome. This study not only contributes to improving the accuracy of prognosis and diagnosis, but also confirms the utility of fNIRS in detecting consciousness in this challenging population. In the future, it is necessary to conduct larger-scale studies to validate these findings and explore the impact of variations in patient condition (such as etiology or chronicity) on fNIRS responses, further determining the clinical applicability of fNIRS in consciousness assessment.

## Data Availability

The raw data supporting the conclusions of this article will be made available by the authors, without undue reservation.
