# Supplementary material for: Detecting cognitive motor dissociation by functional near-infrared spectroscopy
Source: Front Neurol. 2025 Apr 1;16:1532804. doi: 10.3389/fneur.2025.1532804 (PMC11997382; doi:10.3389/fneur.2025.1532804)
Supplement: Supplementary file 1 [file Data_Sheet_1.PDF]

Supplementary Material 1. MNI Coordinates

63 Channels and 48 Optodes

, x, y, z

CH01: 55 -55 52

CH02: 42 -54 63

CH03: 54 44 -9

CH04: 60 28 4

CH05: 40 64 -3

CH06: 15 73 0

CH07: 29 68 13

CH08: -14 74 1

CH09: -36 65 -1

CH10: -24 68 16

CH11: -52 44 -6

CH12: -57 29 7

CH13: -56 -54 51

CH14: -56 -27 56

CH15: -66 -30 41

CH16: 32 -54 72

CH17: 21 -52 76

CH18: 50 51 10

CH19: 56 33 23

CH20: 40 54 26

CH21: 2 68 15

CH22: 16 66 29

CH23: -11 64 31

CH24: -45 53 12

CH25: -35 55 28

CH26: -51 36 24

CH27: -52 -4 55

CH28: -62 -6 40

CH29: -47 21 48

CH30: 34 -29 74

CH31: 19 -6 75

CH32: 21 -30 77

CH33: 32 -7 70

CH34: 50 19 48

CH35: 41 19 58

CH36: -29 -5 70

CH37: -26 20 64

CH38: -41 -4 63

CH39: -39 20 58

CH40: -17 -5 75

CH41: -14 20 68

CH42: 42 -86 -21

CH43: 36 -97 -2

CH44: 57 -31 57

CH45: 45 -30 68

CH46: 55 -6 54

CH47: 43 -6 65

CH48: 68 -31 44

CH49: 65 -6 39

CH50: 17 17 69

CH51: 29 17 65

CH52: -42 -53 63

CH53: -31 -52 72

CH54: -32 -27 74

CH55: -44 -27 68

CH56: -18 -50 76

CH57: -17 -27 78

CH58: 32 -87 -41

CH59: 24 -98 -18

CH60: -27 -97 -19

CH61: -40 -94 -3

CH62: -35 -84 -43

CH63: -44 -81 -25

S1: 48 -66 53

S2: 55 34 -10

S3: 28 69 -1

S4: -25 69 2

S5: -54 34 -8

S6: -62 -41 48

S7: 25 -63 71

S8: 49 45 26

S9: 4 64 29

S10: -44 46 26

S11: -53 6 46

S12: 28 -17 75

S13: 43 29 49

S14: -35 8 65

S15: -12 7 73

S16: 46 -89 -2

S17: 51 -18 63

S18: 69 -18 34

S19: 20 28 62

S20: -38 -41 70

S21: -13 -39 80

S22: 23 -91 -39

S23: -28 -103 -2

S24: -42 -78 -44

D1: 61 -43 51

D2: 48 55 -5

D3: 1 69 0

D4: -44 57 -3

D5: -48 -67 52

D6: 40 -82 -42

D7: 62 21 19

D8: 29 62 26

D9: -22 62 29

D10: -58 24 21

D11: -26 -61 70

D12: 38 -42 70

D13: 26 -104 -1

D14: 14 4 74

D15: -25 -16 75

D16: -26 -89 -40

D17: 58 6 46

D18: -16 32 60

D19: 15 -40 80

D20: 38 7 64

D21: -48 -16 61

D22: -67 -18 33

D23: -39 32 46

D24: -49 -85 -4

Supplementary Material 2. Brodmann Areas

63 Channels and 48 Optodes

\# , Anatomical label, Percentage of Overlap

CH01: 39 - Angular gyrus, part of Wernicke's area, 0.31855

CH01: 40 - Supramarginal gyrus part of Wernicke's area, 0.68145

CH02: 7 - Somatosensory Association Cortex, 0.35124

CH02: 39 - Angular gyrus, part of Wernicke's area, 0.0041322

CH02: 40 - Supramarginal gyrus part of Wernicke's area, 0.64463

CH03: 45 - pars triangularis Broca's area, 0.15088

CH03: 46 - Dorsolateral prefrontal cortex, 0.49474

CH03: 47 - Inferior prefrontal gyrus, 0.35439

CH04: 38 - Temporopolar area, 0.13333

CH04: 44 - pars opercularis, part of Broca's area, 0.022222

CH04: 45 - pars triangularis Broca's area, 0.73968

CH04: 48 - Retrosubicular area, 0.10476

CH05: 10 - Frontopolar area, 0.51321

CH05: 11 - Orbitofrontal area, 0.20377

CH05: 46 - Dorsolateral prefrontal cortex, 0.2

CH05: 47 - Inferior prefrontal gyrus, 0.083019

CH06: 10 - Frontopolar area, 0.63636

CH06: 11 - Orbitofrontal area, 0.36364

CH07: 10 - Frontopolar area, 0.95789

CH07: 11 - Orbitofrontal area, 0.038596

CH07: 46 - Dorsolateral prefrontal cortex, 0.0035088

CH08: 10 - Frontopolar area, 0.71711

CH08: 11 - Orbitofrontal area,   0.28289

CH09: 10 - Frontopolar area,   0.65428
CH09: 11 - Orbitofrontal area,   0.26394
CH09: 46 - Dorsolateral prefrontal cortex,   0.063197
CH09: 47 - Inferior prefrontal gyrus,   0.018587

CH10: 10 - Frontopolar area,   0.96751
CH10: 46 - Dorsolateral prefrontal cortex,   0.032491

CH11: 45 - pars triangularis Broca's area,   0.24823
CH11: 46 - Dorsolateral prefrontal cortex,   0.5461
CH11: 47 - Inferior prefrontal gyrus,   0.20567

CH12: 38 - Temporopolar area,   0.060127
CH12: 44 - pars opercularis, part of Broca's area,   0.028481
CH12: 45 - pars triangularis Broca's area,   0.81962
CH12: 48 - Retrosubicular area,   0.091772

CH13: 39 - Angular gyrus, part of Wernicke's area,   0.22093
CH13: 40 - Supramarginal gyrus part of Wernicke's area,   0.77907

CH14: 1 - Primary Somatosensory Cortex,   0.35294
CH14: 2 - Primary Somatosensory Cortex,   0.066667
CH14: 3 - Primary Somatosensory Cortex,   0.42745
CH14: 40 - Supramarginal gyrus part of Wernicke's area,   0.15294

CH15: 1 - Primary Somatosensory Cortex,   0.13131
CH15: 2 - Primary Somatosensory Cortex,   0.46128
CH15: 3 - Primary Somatosensory Cortex,   0.013468
CH15: 40 - Supramarginal gyrus part of Wernicke's area,   0.39394

CH16: 1 - Primary Somatosensory Cortex,   0.010989
CH16: 5 - Somatosensory Association Cortex,   0.032967

CH16: 7 - Somatosensory Association Cortex,   0.95604

CH17: 1 - Primary Somatosensory Cortex,   0.10738
CH17: 5 - Somatosensory Association Cortex,   0.43624
CH17: 7 - Somatosensory Association Cortex,   0.45638

CH18: 45 - pars triangularis Broca's area,   0.20149
CH18: 46 - Dorsolateral prefrontal cortex,   0.79851

CH19: 45 - pars triangularis Broca's area,   1

CH20: 45 - pars triangularis Broca's area,   0.10573
CH20: 46 - Dorsolateral prefrontal cortex,   0.89427

CH21: 10 - Frontopolar area,   1

CH22: 9 - Dorsolateral prefrontal cortex,   0.17518
CH22: 10 - Frontopolar area,   0.82482

CH23: 9 - Dorsolateral prefrontal cortex,   0.2695
CH23: 10 - Frontopolar area,   0.7305

CH24: 10 - Frontopolar area,   0.042802
CH24: 45 - pars triangularis Broca's area,   0.1284
CH24: 46 - Dorsolateral prefrontal cortex,   0.82879

CH25: 46 - Dorsolateral prefrontal cortex,   1

CH26: 45 - pars triangularis Broca's area,   1

CH27: 4 - Primary Motor Cortex,   0.19841
CH27: 6 - Pre-Motor and Supplementary Motor Cortex,   0.80159

CH28: 1 - Primary Somatosensory Cortex,   0.079585

CH28: 3 - Primary Somatosensory Cortex,   0.14187

CH28: 4 - Primary Motor Cortex,   0.28028

CH28: 6 - Pre-Motor and Supplementary Motor Cortex,   0.22145

CH28: 43 - Subcentral area,   0.27682

CH29: 6 - Pre-Motor and Supplementary Motor Cortex,   0.0080972

CH29: 9 - Dorsolateral prefrontal cortex,   0.7166

CH29: 44 - pars opercularis, part of Broca's area,   0.2753

CH30: 3 - Primary Somatosensory Cortex,   0.19868

CH30: 4 - Primary Motor Cortex,   0.71854

CH30: 6 - Pre-Motor and Supplementary Motor Cortex,   0.082781

CH31: 6 - Pre-Motor and Supplementary Motor Cortex,   1

CH32: 3 - Primary Somatosensory Cortex,   0.090909

CH32: 4 - Primary Motor Cortex,   0.80519

CH32: 6 - Pre-Motor and Supplementary Motor Cortex,   0.1039

CH33: 6 - Pre-Motor and Supplementary Motor Cortex,   1

CH34: 6 - Pre-Motor and Supplementary Motor Cortex,   0.047244

CH34: 9 - Dorsolateral prefrontal cortex,   0.72835

CH34: 44 - pars opercularis, part of Broca's area,   0.22441

CH35: 6 - Pre-Motor and Supplementary Motor Cortex,   0.040816

CH35: 8 - Includes Frontal eye fields,   0.26122

CH35: 9 - Dorsolateral prefrontal cortex,   0.69796

CH36: 6 - Pre-Motor and Supplementary Motor Cortex,   1

CH37: 6 - Pre-Motor and Supplementary Motor Cortex,   0.099631

CH37: 8 - Includes Frontal eye fields,   0.89299

CH37: 9 - Dorsolateral prefrontal cortex,   0.0073801

CH38: 4 - Primary Motor Cortex,   0.10305

CH38: 6 - Pre-Motor and Supplementary Motor Cortex,   0.89695

CH39: 6 - Pre-Motor and Supplementary Motor Cortex,   0.089069

CH39: 8 - Includes Frontal eye fields,   0.31579

CH39: 9 - Dorsolateral prefrontal cortex,   0.59514

CH40: 6 - Pre-Motor and Supplementary Motor Cortex,   1

CH41: 6 - Pre-Motor and Supplementary Motor Cortex,   0.44483

CH41: 8 - Includes Frontal eye fields,   0.55517

CH42: 18 - Visual Association Cortex (V2),   0.039526

CH42: 19 - V3,   0.96047

CH43: 17 - Primary Visual Cortex (V1),   0.11027

CH43: 18 - Visual Association Cortex (V2),   0.88973

CH44: 1 - Primary Somatosensory Cortex,   0.48679

CH44: 2 - Primary Somatosensory Cortex,   0.079245

CH44: 3 - Primary Somatosensory Cortex,   0.12453

CH44: 40 - Supramarginal gyrus part of Wernicke's area,   0.30943

CH45: 1 - Primary Somatosensory Cortex,   0.1434

CH45: 2 - Primary Somatosensory Cortex,   0.022642

CH45: 3 - Primary Somatosensory Cortex,   0.4566

CH45: 4 - Primary Motor Cortex,   0.37736

CH46: 4 - Primary Motor Cortex,   0.34677

CH46: 6 - Pre-Motor and Supplementary Motor Cortex,   0.65323

CH47: 4 - Primary Motor Cortex,   0.26923

CH47: 6 - Pre-Motor and Supplementary Motor Cortex,   0.73077

CH48: 1 - Primary Somatosensory Cortex,   0.23569

CH48: 2 - Primary Somatosensory Cortex,   0.20875

CH48: 40 - Supramarginal gyrus part of Wernicke's area,   0.55556

CH49: 1 - Primary Somatosensory Cortex,   0.090909

CH49: 3 - Primary Somatosensory Cortex,   0.14685

CH49: 4 - Primary Motor Cortex,   0.25524

CH49: 6 - Pre-Motor and Supplementary Motor Cortex,   0.15734

CH49: 43 - Subcentral area,   0.34965

CH50: 6 - Pre-Motor and Supplementary Motor Cortex,   0.6263

CH50: 8 - Includes Frontal eye fields,   0.3737

CH51: 6 - Pre-Motor and Supplementary Motor Cortex,   0.11913

CH51: 8 - Includes Frontal eye fields,   0.85921

CH51: 9 - Dorsolateral prefrontal cortex,   0.021661

CH52: 2 - Primary Somatosensory Cortex,   0.056452

CH52: 7 - Somatosensory Association Cortex,   0.21774

CH52: 39 - Angular gyrus, part of Wernicke's area,   0.040323

CH52: 40 - Supramarginal gyrus part of Wernicke's area,   0.68548

CH53: 1 - Primary Somatosensory Cortex,   0.10791

CH53: 5 - Somatosensory Association Cortex,   0.014388

CH53: 7 - Somatosensory Association Cortex,   0.85971

CH53: 40 - Supramarginal gyrus part of Wernicke's area,   0.017986

CH54: 3 - Primary Somatosensory Cortex,   0.090615

CH54: 4 - Primary Motor Cortex,   0.75081

CH54: 6 - Pre-Motor and Supplementary Motor Cortex,   0.15858

CH55: 1 - Primary Somatosensory Cortex,   0.15018

CH55: 3 - Primary Somatosensory Cortex,   0.42125

CH55: 4 - Primary Motor Cortex,   0.42857

CH56: 1 - Primary Somatosensory Cortex,   0.20401
CH56: 3 - Primary Somatosensory Cortex,   0.013378
CH56: 5 - Somatosensory Association Cortex,   0.45151
CH56: 7 - Somatosensory Association Cortex,   0.3311

CH57: 3 - Primary Somatosensory Cortex,   0.0094044
CH57: 4 - Primary Motor Cortex,   0.8652
CH57: 6 - Pre-Motor and Supplementary Motor Cortex,   0.12539

CH58: 18 - Visual Association Cortex (V2),   0.017921
CH58: 19 - V3,   0.98208

CH59: 17 - Primary Visual Cortex (V1),   0.0037175
CH59: 18 - Visual Association Cortex (V2),   0.99628

CH60: 18 - Visual Association Cortex (V2),   0.95038
CH60: 19 - V3,   0.049618

CH61: 18 - Visual Association Cortex (V2),   0.75197
CH61: 19 - V3,   0.24803

CH62: 19 - V3,   1

CH63: 19 - V3,   1

S1: 7 - Somatosensory Association Cortex,   0.22422
S1: 39 - Angular gyrus, part of Wernicke's area,   0.68161
S1: 40 - Supramarginal gyrus part of Wernicke's area,   0.09417

S2: 38 - Temporopolar area,   0.31359
S2: 45 - pars triangularis Broca's area,   0.27526
S2: 46 - Dorsolateral prefrontal cortex,   0.097561

S2: 47 - Inferior prefrontal gyrus, 0.31359

S3: 10 - Frontopolar area, 0.32026
S3: 11 - Orbitofrontal area, 0.67974

S4: 10 - Frontopolar area, 0.56478
S4: 11 - Orbitofrontal area, 0.43522

S5: 38 - Temporopolar area, 0.28814
S5: 45 - pars triangularis Broca's area, 0.38644
S5: 46 - Dorsolateral prefrontal cortex, 0.081356
S5: 47 - Inferior prefrontal gyrus, 0.24407

S6: 40 - Supramarginal gyrus part of Wernicke's area, 1

S7: 5 - Somatosensory Association Cortex, 0.0039216
S7: 7 - Somatosensory Association Cortex, 0.99608

S8: 45 - pars triangularis Broca's area, 0.76829
S8: 46 - Dorsolateral prefrontal cortex, 0.23171

S9: 9 - Dorsolateral prefrontal cortex, 0.121
S9: 10 - Frontopolar area, 0.879

S10: 45 - pars triangularis Broca's area, 0.56904
S10: 46 - Dorsolateral prefrontal cortex, 0.43096

S11: 4 - Primary Motor Cortex, 0.003876
S11: 6 - Pre-Motor and Supplementary Motor Cortex, 0.77907
S11: 9 - Dorsolateral prefrontal cortex, 0.15891
S11: 44 - pars opercularis, part of Broca's area, 0.05814

S12: 4 - Primary Motor Cortex, 0.15842
S12: 6 - Pre-Motor and Supplementary Motor Cortex, 0.84158

S13: 8 - Includes Frontal eye fields,   0.0088106

S13: 9 - Dorsolateral prefrontal cortex,   0.92952

S13: 44 - pars opercularis, part of Broca's area,   0.052863

S13: 45 - pars triangularis Broca's area,   0.0088106

S14: 6 - Pre-Motor and Supplementary Motor Cortex,   0.66065

S14: 8 - Includes Frontal eye fields,   0.28881

S14: 9 - Dorsolateral prefrontal cortex,   0.050542

S15: 6 - Pre-Motor and Supplementary Motor Cortex,   1

S16: 18 - Visual Association Cortex (V2),   0.41079

S16: 19 - V3,   0.58921

S17: 1 - Primary Somatosensory Cortex,   0.03629

S17: 3 - Primary Somatosensory Cortex,   0.41129

S17: 4 - Primary Motor Cortex,   0.55242

S18: 1 - Primary Somatosensory Cortex,   0.25

S18: 2 - Primary Somatosensory Cortex,   0.53481

S18: 43 - Subcentral area,   0.21519

S19: 8 - Includes Frontal eye fields,   1

S20: 1 - Primary Somatosensory Cortex,   0.27174

S20: 2 - Primary Somatosensory Cortex,   0.2029

S20: 3 - Primary Somatosensory Cortex,   0.25725

S20: 4 - Primary Motor Cortex,   0.083333

S20: 7 - Somatosensory Association Cortex,   0.1558

S20: 40 - Supramarginal gyrus part of Wernicke's area,   0.028986

S21: 1 - Primary Somatosensory Cortex,   0.15858

S21: 3 - Primary Somatosensory Cortex,   0.19094

S21: 4 - Primary Motor Cortex, 0.40453
S21: 5 - Somatosensory Association Cortex, 0.24595

S22: 18 - Visual Association Cortex (V2), 0.59524
S22: 19 - V3, 0.40476

S23: 17 - Primary Visual Cortex (V1), 0.5784
S23: 18 - Visual Association Cortex (V2), 0.4216

S24: 19 - V3, 1

D1: 40 - Supramarginal gyrus part of Wernicke's area, 1

D2: 10 - Frontopolar area, 0.094862
D2: 46 - Dorsolateral prefrontal cortex, 0.81423
D2: 47 - Inferior prefrontal gyrus, 0.090909

D3: 10 - Frontopolar area, 0.93919
D3: 11 - Orbitofrontal area, 0.060811

D4: 10 - Frontopolar area, 0.24
D4: 46 - Dorsolateral prefrontal cortex, 0.728
D4: 47 - Inferior prefrontal gyrus, 0.032

D5: 7 - Somatosensory Association Cortex, 0.11667
D5: 39 - Angular gyrus, part of Wernicke's area, 0.80417
D5: 40 - Supramarginal gyrus part of Wernicke's area, 0.079167

D6: 19 - V3, 1

D7: 6 - Pre-Motor and Supplementary Motor Cortex, 0.091803
D7: 44 - pars opercularis, part of Broca's area, 0.47213
D7: 45 - pars triangularis Broca's area, 0.43607

D8: 9 - Dorsolateral prefrontal cortex,   0.023904
D8: 10 - Frontopolar area,   0.4741
D8: 46 - Dorsolateral prefrontal cortex,   0.50199

D9: 9 - Dorsolateral prefrontal cortex,   0.19048
D9: 10 - Frontopolar area,   0.45635
D9: 46 - Dorsolateral prefrontal cortex,   0.35317

D10: 44 - pars opercularis, part of Broca's area,   0.4339
D10: 45 - pars triangularis Broca's area,   0.5661

D11: 5 - Somatosensory Association Cortex,   0.0037037
D11: 7 - Somatosensory Association Cortex,   0.9963

D12: 1 - Primary Somatosensory Cortex,   0.25092
D12: 2 - Primary Somatosensory Cortex,   0.13284
D12: 3 - Primary Somatosensory Cortex,   0.26937
D12: 4 - Primary Motor Cortex,   0.051661
D12: 7 - Somatosensory Association Cortex,   0.22878
D12: 40 - Supramarginal gyrus part of Wernicke's area,   0.066421

D13: 17 - Primary Visual Cortex (V1),   0.76271
D13: 18 - Visual Association Cortex (V2),   0.23729

D14: 6 - Pre-Motor and Supplementary Motor Cortex,   1

D15: 4 - Primary Motor Cortex,   0.16066
D15: 6 - Pre-Motor and Supplementary Motor Cortex,   0.83934

D16: 18 - Visual Association Cortex (V2),   0.2
D16: 19 - V3,   0.8

D17: 4 - Primary Motor Cortex,   0.015748
D17: 6 - Pre-Motor and Supplementary Motor Cortex,   0.88189

D17: 9 - Dorsolateral prefrontal cortex,   0.066929
D17: 44 - pars opercularis, part of Broca's area,   0.035433

D18: 8 - Includes Frontal eye fields,   0.96241
D18: 9 - Dorsolateral prefrontal cortex,   0.037594

D19: 1 - Primary Somatosensory Cortex,   0.14826
D19: 3 - Primary Somatosensory Cortex,   0.19558
D19: 4 - Primary Motor Cortex,   0.35647
D19: 5 - Somatosensory Association Cortex,   0.29968

D20: 6 - Pre-Motor and Supplementary Motor Cortex,   0.63636
D20: 8 - Includes Frontal eye fields,   0.24364
D20: 9 - Dorsolateral prefrontal cortex,   0.12

D21: 3 - Primary Somatosensory Cortex,   0.31513
D21: 4 - Primary Motor Cortex,   0.57983
D21: 6 - Pre-Motor and Supplementary Motor Cortex,   0.10504

D22: 1 - Primary Somatosensory Cortex,   0.23418
D22: 2 - Primary Somatosensory Cortex,   0.46519
D22: 3 - Primary Somatosensory Cortex,   0.0031646
D22: 43 - Subcentral area,   0.25633
D22: 48 - Retrosubicular area,   0.041139

D23: 9 - Dorsolateral prefrontal cortex,   0.85388
D23: 44 - pars opercularis, part of Broca's area,   0.027397
D23: 45 - pars triangularis Broca's area,   0.045662
D23: 46 - Dorsolateral prefrontal cortex,   0.073059

D24: 18 - Visual Association Cortex (V2),   0.026923
D24: 19 - V3,   0.97308
