# Supplementary material for: Detecting cognitive motor dissociation by functional near-infrared spectroscopy
Source: Front Neurol. 2025 Apr 1;16:1532804. doi: 10.3389/fneur.2025.1532804 (PMC11997382; doi:10.3389/fneur.2025.1532804)
Supplement: Supplementary file 2 [file Data_Sheet_2.PDF]

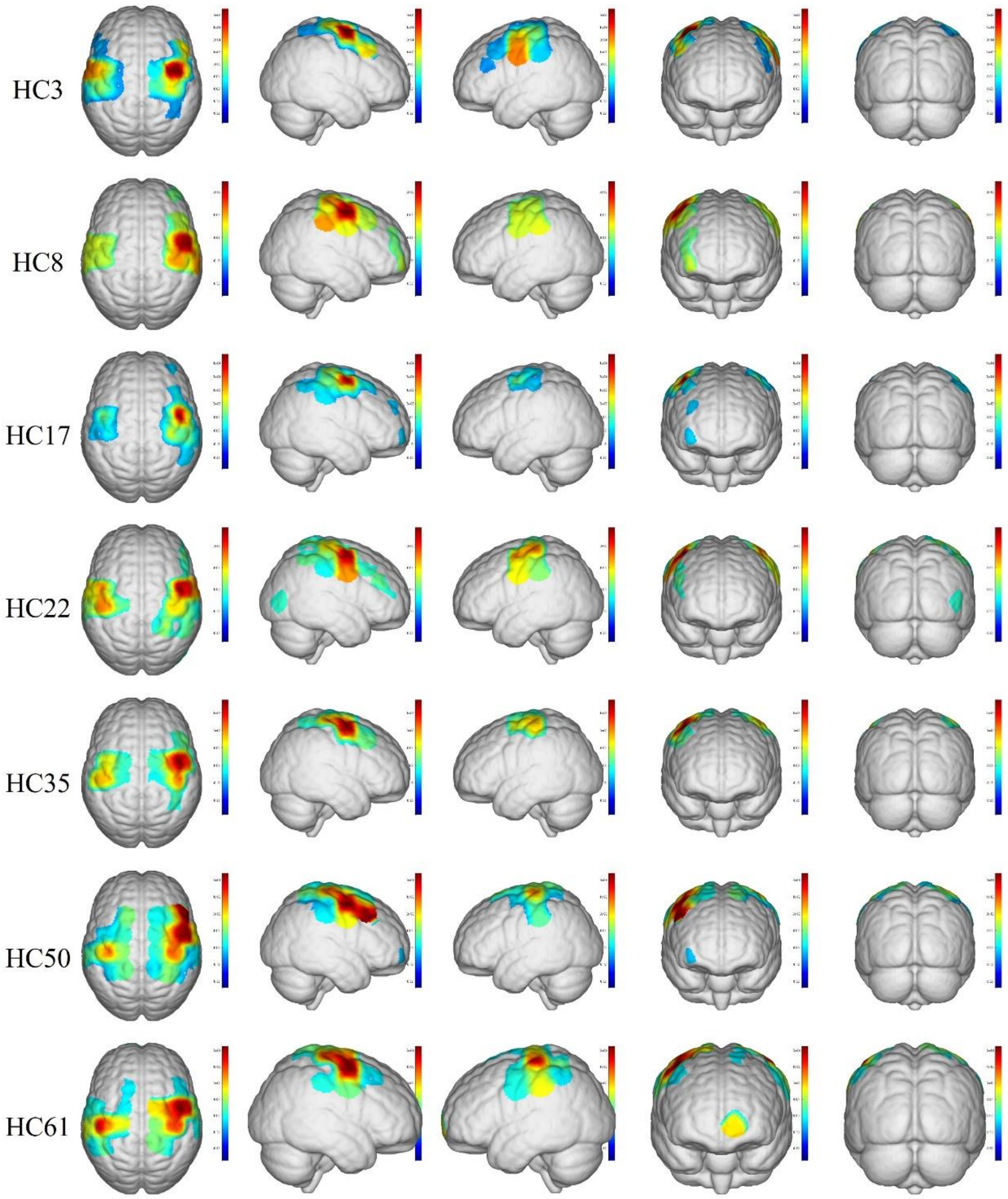

Supplementary Figure 1. Brain activation maps of the motor imagery (MI) task for 7 healthy controls (HC). The activated channels were identified using the general linear model and primarily concentrated in bilateral motor-related brain regions. Higher color scales indicate a greater level of activation.
